# Investigation on the nasal airflow characteristics of anterior nasal cavity stenosis

**DOI:** 10.1590/1414-431X20165182

**Published:** 2016-08-01

**Authors:** T. Wang, D. Chen, P.H. Wang, J. Chen, J. Deng

**Affiliations:** 1 Department of Otorhinolaryngology, Shanghai Ninth People's Hospital, Shanghai Jiao Tong University School of Medicine, Shanghai, China; 2 Institute of Aeronautics and Astronautics, Zhejiang University, Hangzhou, Zhejiang, China

**Keywords:** Anterior nasal cavity stenosis, Computational fluid dynamics, Nasal valve area, Airflow characteristics

## Abstract

We used a computational fluid dynamics (CFD) model to study the inspiratory airflow profiles of patients with anterior nasal cavity stenosis who underwent curative surgery, by comparing pre- and postoperative airflow characteristics. Twenty patients with severe anterior nasal cavity stenosis, including one case of bilateral stenosis, underwent computed tomography (CT) scans for CFD modelling. The pre- and postoperative airflow characteristics of the nasal cavity were simulated and analyzed. The narrowest area of the nasal cavity in all 20 patients was located within the nasal valve area, and the mean cross-sectional area increased from 0.39 cm^2^ preoperative to 0.78 cm^2^ postoperative (P<0.01). Meanwhile, the mean airflow velocity in the nasal valve area decreased from 6.19 m/s to 2.88 m/s (P<0.01). Surgical restoration of the nasal symmetry in the bilateral nasal cavity reduced nasal resistance in the narrow sides from 0.24 Pa^.^s/mL to 0.11 Pa^.^s/mL (P<0.01). Numerical simulation of the nasal cavity in patients with anterior nasal cavity stenosis revealed structural changes and the resultant patterns of nasal airflow. Surgery achieved balanced bilateral nasal ventilation and decreased nasal resistance in the narrow region of the nasal cavity. The correction of nasal valve stenosis is not only indispensable for reducing nasal resistance, but also the key to obtain satisfactory curative effect.

## Introduction

The nasal cavity, an important passage of the upper respiratory tract, has various functions including cleaning, humidifying and warming the inhaled air. Anterior nasal cavity stenosis, which occurs in the anterior nostril, nasal valve area or around nasal vestibule, can lead to nasal ventilation dysfunction and structural deformities. Stenosis can also occur in the nasal vestibule and postnaris after trauma, infection, or surgery. Ulceration secondary to infection, burns, tumor resection, nasal intubation or radiotherapy may also lead to rhinostenosis. Scar stenosis is frequently seen in the nasal valve area due to the weakness of the cartilaginous supporting structures. Hence, surgical treatment methods should be chosen according to the location of stenosis and the thickness of local tissue. The main surgical procedures include: a) scar resection, b) intranasal Z-plasty, and c) nasal stenting (silicone tube) in the anterior nasal cavity to prevent recurrent stenosis.

Much research has examined outcomes of various surgical techniques used to correct anterior nasal cavity stenosis. However, the airflow profiles of anterior nasal cavity stenosis and their correlation with clinical symptoms have not been well investigated due to the anatomical complexity of the nasal cavity. In recent years, with the development of computational fluid dynamics (CFD) and biological numerical simulation methods, many researchers have employed these methods to study nasal physiological function and the influence of nasal structure change on the airflow distribution (1) and nasal heating function (2–4).

Most studies to date have shown that CFD can simulate the basic features of nasal physiological function with a given geometry. CFD studies on nasal anomalies have also been useful for diagnosis, evaluating degree of dysfunction, and treatment prediction including surgical planning and evaluating outcomes of surgical intervention (5,6).

Our previous studies focusing on numerical simulation analysis of the pre- and postoperative nasal cavity in Crouzon syndrome have verified the reliability and accuracy of CFD for analyzing nasal airflow features (7). Here, we created CFD models for the nasal cavity of patients with anterior nasal cavity stenosis and assessed the aerodynamics of the nasal cavity during the inspiratory phase. Additionally, we compared pre- and postoperative airflow patterns of the nasal cavity and evaluated the impact of nasal ventilation function after surgery.

## Subjects and Methods

### Subjects

This study included 20 patients (15 males and 5 females) with anterior nasal cavity stenosis (1 female had bilateral stenosis) who were diagnosed between January 2014 and May 2015. The age range was 29 to 46 years. The causes of stenosis were trauma related to car accidents in 16 patients, injury due to nasal intubation in 2 patients and nasal vestibule infection in 2 patients. None of the subjects was suffering from any nasal inflammation, polyposis or previous nasal surgery. All subjects had undergone surgery including nasal vestibule scar excision, local skin flap transplantation and the use of postoperative intranasal stenting (silicone tube).

### Ethical considerations

The study protocol was approved by the Ethics Committees of Shanghai Ninth People's Hospital (approval #201478). All participants provided written informed consent.

### Research method

A 64-slice spiral computed tomograph (CT; LightSpeed Ultra from GE Healthcare, USA) was used for scanning. The patients' nasal cavity was cleaned prior to CT scan. The patients remained quiet in a supine position at constant room temperature. The scan parameters were 120 mA, 120 kV, thread pitch of 1.375 mm, and velocity of 27.5 mm/s, while the parameters of three-dimensional reconstruction were set to a resolution of 512×512 pixels, slice thickness of 0.75 mm, window width of 2000 Hounsfield units (Hu), and window level of 200 Hu.

### Establishment of the three-dimensional CFD model of the nasal cavity

Three-dimensional CFD model of the nasal airway was reconstructed from 100 axial images of each patient's nasal cavity obtained by thin-layer CT scanning. The image segmentation was performed using the medical imaging software, MIMICS (Mimics Research 17.0 for X64 Platform 17.0.0.435: Materialise N.V., Technologielaan 15, BE-3001, Belgium), and the airway was identified in each of the axial images based on a predefined threshold set between -1024 and -200 HU. The three-dimensional raw model was then exported into a remeshing software, 3-matic Research 9.0 (Materialise N.V.) to demarcate the individual surfaces of the airway and optimize the surface mesh quality for successful CFD modeling.

### CFD analysis

Due to the complex geometry of the nasal cavity, a computational software for fluid dynamics, FLUENT 6.3.26 (ANSYS Inc., USA) was used to perform numerical simulations that enable the prediction of flow field within the model. The boundary conditions for the simulations were input as follows: assuming the entire nasal mucosa to be a solid wall, the fluid was the air (ρ=1.225 kg/m^3^, dynamic viscosity coefficient μ=1.7894×10^-5^ kg·m^-1^·s^-1^), which was set as the steady flow of an incompressible fluid. During numerical simulation, the influence of temperature and humidity changes was neglected. A no-slip boundary was applied at the nasal wall and the computational region extended from the anterior nostril to the nasopharynx. Standard atmospheric pressure was set at 0 Pa. The pressure inlet and velocity outlet boundary conditions were set at the anterior nostril entrance and nasopharynx, respectively. Constant flow was set at 400 mL/s. The Navier-Stokes equation (continuity equation for an incompressible fluid) and laminar flow model were applied to compute the airway flow characteristics (8,9). Numerical simulations provided detailed airflow parameters, including pressure distribution chart, velocity vector chart and airflow diagram. Nasal resistance was expressed as Rn=Pn/Vn (10), where Rn is the nasal resistance, Pn is the pressure difference of the entire nasal cavity, and Vn is the airflow rate.

### Statistical analyses

After the numerical simulation, statistical analyses of the airflow field data, including pressure and velocity, were performed using a specialized statistical software (SAS release 8.01 TS Level 01M0). The comparisons of pressure and velocity in the appointed pre- and postoperative cross-sections were analyzed by paired *t*-tests, while comparisons of pressure and velocity on the narrow and non-narrow sides of the nasal cavity were made using independent samples *t*-tests. P<0.05 were considered to be statistically significant. The relationship between unilateral nasal resistance and velocity and cross-sectional area at the nasal valve on the narrow side was analyzed by Curve Estimation.

## Results

### Anterior nasal cavity stenosis features

**Figure 1:**
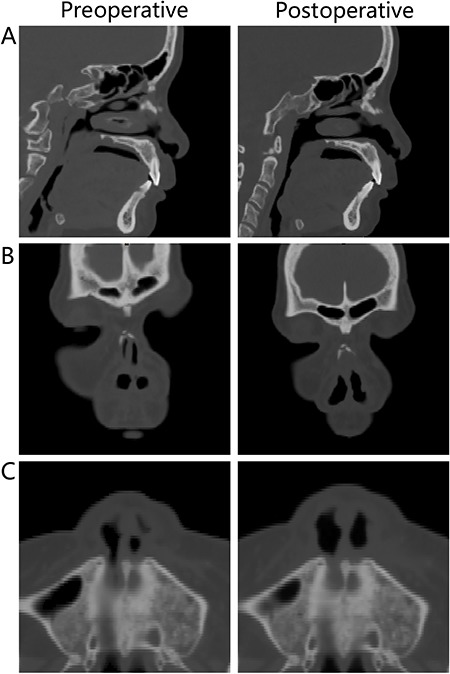
Pre- and postoperative anterior nasal cavity stenosis. *A*, sagittal and *B*, coronal computed tomography of the nasal cavity. *C*, Nasal base view.

**Figure 2:**
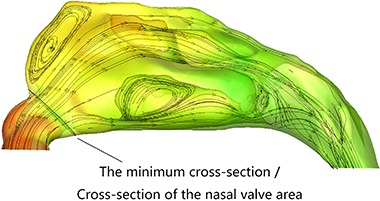
Transversal section of the nasal cavity. Circled area represents the minimum/total cross-section of the nasal valve area. The arrows show the air flow direction.

The nasal cavity CFD models indicated that the anterior nasal cavity stenoses of the 20 patients were all located in the nasal valve area with a mean cross-sectional area of 0.39±0.16 cm^2^ (compared to 0.85±0.2 cm^2^ in the nasal valve area on the non-narrow side). Postoperatively, the normal anatomy of nasal valve was restored (Figure 1). The minimum/total cross-section of the nasal valve area ratio (Figure 2) was identified perpendicular to the airflow direction by visual inspection; the minimum cross-sectional area of the narrow side was increased postoperatively (mean=0.78±0.18 cm^2^; P<0.0001; Table 1).

**Table:**
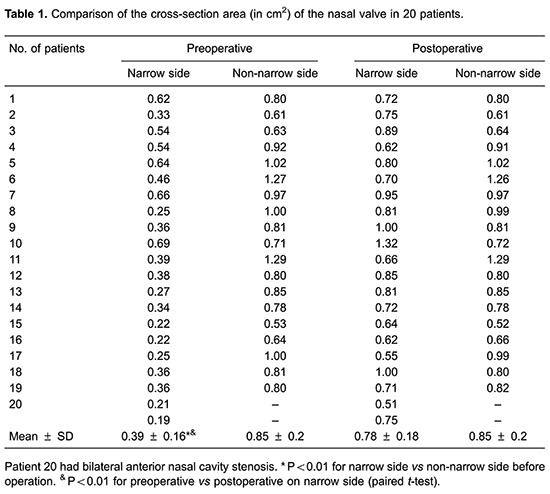

### Preoperative pressure and velocity in the inspiratory phase

**Figure 3:**
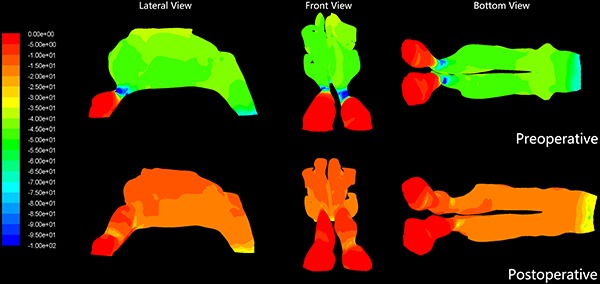
Pre- and postoperative airflow pressure distribution of the nasal cavity in one patient with anterior nasal cavity stenosis. Comparison of pre- and postoperative pressure distribution revealed that the pressure gradually decreased from the anterior nostril to the nasopharynx. Red color indicates higher pressure.

**Figure 4:**
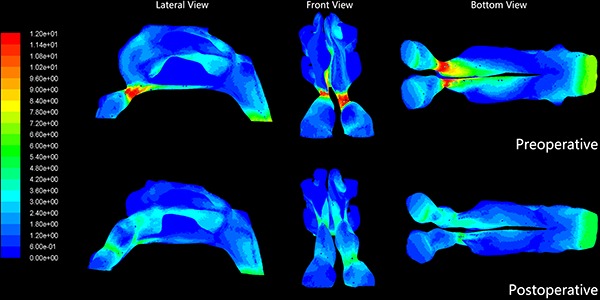
Pre- and postoperative airflow velocity distributions of the nasal cavity in one patient with anterior nasal cavity stenosis. Comparison of pre- and postoperative velocity distribution revealed that the largest velocity appeared in the nasal valve area. Red color indicates higher velocity.

Figures 3 and 4 show the distributions of pressure and velocity during steady-state inspiratory airflow, respectively. The maximum pressure appears in the nasal valve area preoperatively (Figure 3). Most airway resistance was located from the anterior part of the nasal cavity, namely the anterior naris, to the anterior portion of inferior turbinate, where the pressure dropped dramatically. In the narrow side of the nasal cavity, the pressure on the front-end plane of the inferior turbinate accounted for 32–91% of the total pressure in the nasal cavity (mean value= 8.6±18.3%). Meanwhile, in the non-narrow side of nasal cavity, the preoperative pressure accounted for only 23.3±10.9% of the total pressure in the nasal cavity (Table 2). In the 21 narrow sides of the nasal cavity models, stenosis were also found within the nasal valve area. Mean pressure on the cross-section of the nasal valve area was -37.3±23.2 Pa in the narrow side of nasal cavity *vs* -8.1±3.4 Pa in the non-narrow side of nasal cavity (P<0.01; Table 3).

**Table:**
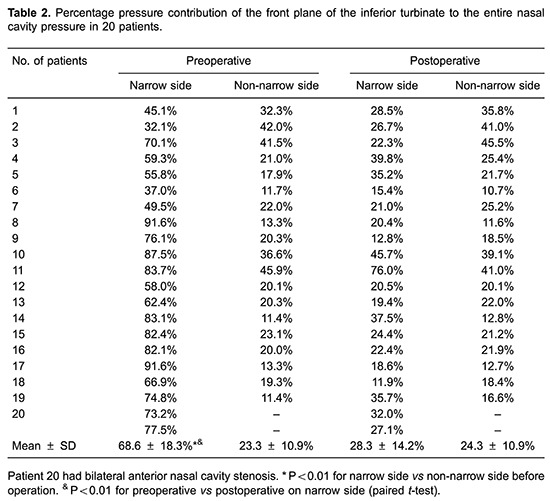

**Table:**
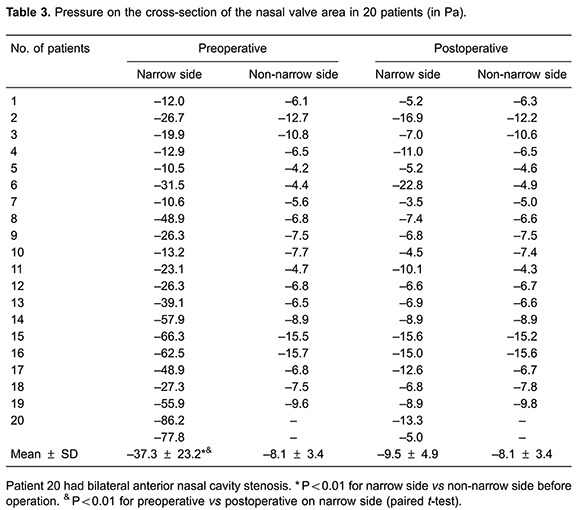

The velocity distribution charts in Figure 4 show that the maximum velocities were also found in the nasal valve area. The mean maximum velocity was 6.19 ± 2.3 m/s in the 21 narrow sides and 2.57±0.6 m/s in the 19 non-narrow sides (P<0.01; Table 4).

**Table:**
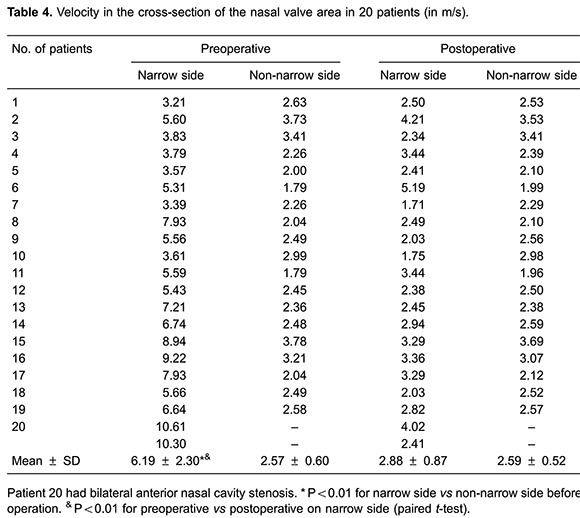

### Postoperative pressure and velocity in the inspiratory phase

In the inspiratory phase, differential pressure (Δpsid) between the anterior nostril inlet and the nasopharynx outlet significantly decreased postoperatively (P<0.01). There was no significant difference in Δpsid postoperative between narrow and non-narrow sides of the nasal cavity (P=0.116>0.05; Table 5). The percentage pressure contribution of the front plane of the inferior turbinate to the entire nasal cavity pressure decreased significantly. The mean value decreased from 68.6±18.3% to 28.3±14.2%, postoperative (P<0.01; Table 2). Pressure on the nasal valve area was -37.3 ± 23.2 Pa preoperative *vs* -9.5±4.9 Pa postoperative (P<0.01; Table 3). The preoperative mean velocity of the nasal valve area (6.19±2.30 m/s) was significantly higher than the postoperative mean velocity (2.88±0.87 m/s; P<0.01; Table 4).

**Table:**
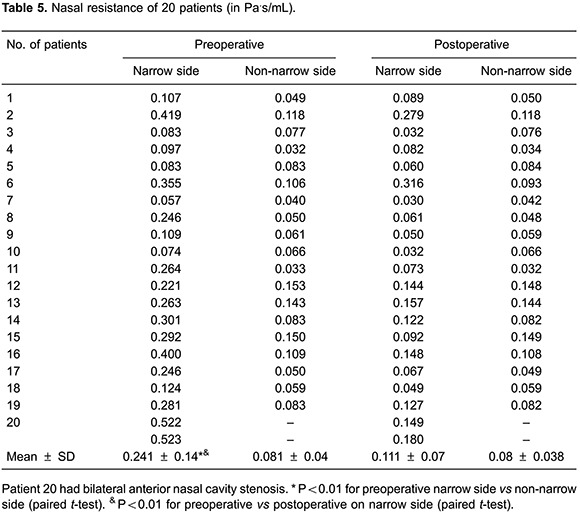

### Pressure and velocity gradient in the inspiratory phase

**Figure 5:**
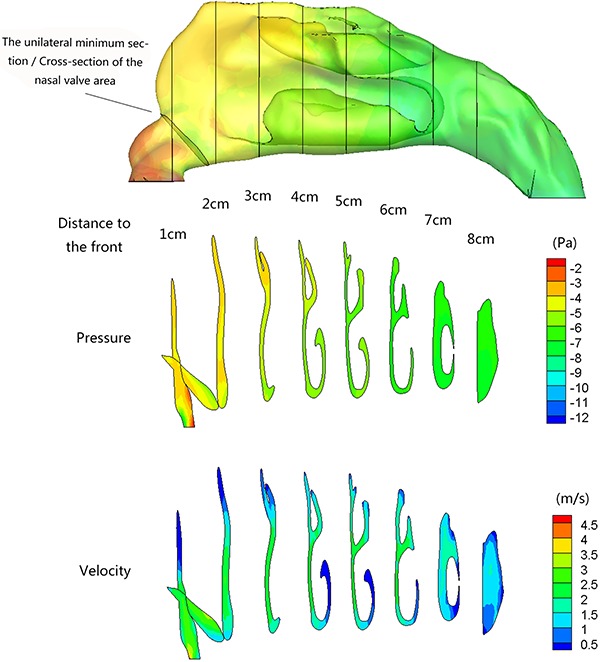
Representative lateral view cross-section showing the pressure and velocity gradient distribution from nostril to nasopharynx.

To facilitate analysis, bilateral coronal cross-sections were created perpendicular to the nasal floor. Starting at the front end of every nasal vestibule, the cross-section intersected at every 1 cm and measured as 1, 2, or 3 cm (Figure 5). This was followed by an analysis on the representative sections of a list of parameters which consisted of cross-sectional area, pressure, velocity, and others. With increasing distance from the nostril, the pressure and velocity in the nasal cavity decreased gradually. Pressure on the front region of the inferior turbinate accounted for 68.6±18.3% of the entire nasal cavity in the narrow side, and 23.3±10.9% in the non-narrow side. Meanwhile, the mean velocity of the different cross-sections showed that the velocity at the nasal valve area was relatively higher and tended to decrease with increasing space.

## Discussion

The influence of the nasal cavity structure on nasal function has been widely studied recently. CFD is a very sophisticated technique for studying and analyzing the airflow features of nasal cavity. Lu et al. (11) reported that the numerical simulation model provides an accurate reflection of nasal airflow, and results obtained by numerical simulation were consistent with clinical measurements. Abouali and coworkers (12) previously used the CFD method to study the airflow of nasal cavity and evaluate the effect of functional endoscopic sinus surgery on nasal cavity airflow. Garcia et al. (13) and Lee et al. (14) also used CFD to study the airflow features of the nasal cavity with pathological structures (inferior turbinate hypertrophy and atrophic rhinitis) and investigate the effect of postoperative structural change on its airflow.

The CFD analysis and numerical simulation model demonstrated good correlation. In our study, the major structure of the nasal cavity model was not simplified. To improve the precision of numerical simulation, the data used in this experiment were obtained by spiral 64-slice CTs with a higher-density resolution for better imaging of the nasal cavity structure. The model was set to rigid body dynamics to omit the effect of respiratory-related soft-tissue deformation which often involves fluid flow and structural solid coupling. Previous reports have shown that the model which was affected by fluid-solid coupling, was generally simplified (9). Zang H et al. (3) claimed that the normal nasal cavity has a certain heating and humidifying effect. However, through the inspection of the Grashof number, Prandtl number, as well as the analysis of heat transfer, the authors concluded that temperature and humidity had no significant effect on the internal nasal airflow under normal breathing conditions. As such, the temperature change in the nasal cavity was not taken into account in this study.

In the current study, we set the internal airflow of the nasal airway to a steady state. In the literature (15), a study on the nasal cavity airflow under quiet respiration (7.5–12 L/min), was conducted in a laminar flow where little turbulence was present. Some researchers (16) concluded that air flowed smoothly in the nasal cavity under normal breathing. The Strouhal number (ωL /U≅0.18) also indicated that the equation, which indicated that internal nasal airflow was quasi-steady, is reasonable and feasible. The results of this study showed that the distribution of pressure and velocity in the nasal cavity were consistent with the conclusions of several relevant reports (17–19).

### Preoperative structure and airflow of anterior nasal cavity stenosis

**Figure 6:**
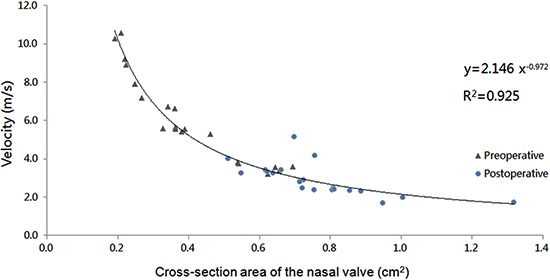
Pre- and postoperative unilateral velocity *vs* cross-sectional area at the nasal valve on the narrow side of nasal cavity.

All patients in this study had anterior nasal cavity stenosis within the nasal valve area. An abnormal stenosis at the front of the nasal cavity may cause a subjective ventilation disorder. When numerical simulation was performed at a constant flow rate (400 mL/s) in both sides of the nasal cavity to ensure constant airflow through the nasal stenosis area, there was an increase in the pressure difference at both ends of the nasal stenosis area. A greater regional pressure difference indicated a higher local nasal resistance. From clinical viewpoints, we deduced that an increase in nasal resistance is mainly attributed by nasal obstructions caused by the narrowest area of the nasal cavity. From the pressure distribution diagram, it can be inferred that the pressure drop is mainly confined to the anterior segment of the nasal cavity, especially within the nasal valve area. From our data, it is likely that the area in front of the inferior turbinate is the narrowest area of the nasal cavity to confer nasal resistance. Results revealed that the nasal valve area had maximum velocity, and the velocity on the narrow side of the nasal cavity was higher than that on the non-narrow side. It can be inferred that the velocity is inversely proportional to the cross-sectional area (correlation coefficient R^2^=0.925; Figure 6).

### Effect of nasal cavity structural change on airflow

Our study included a patient with bilateral anterior nasal cavity stenosis, who underwent bilateral surgery and was used only for the analysis of pre- and postoperative conditions, with no contralateral comparison. Our results of the CFD numerical simulation after CT scanning showed that the nasal valve area increased greatly.

**Figure 7:**
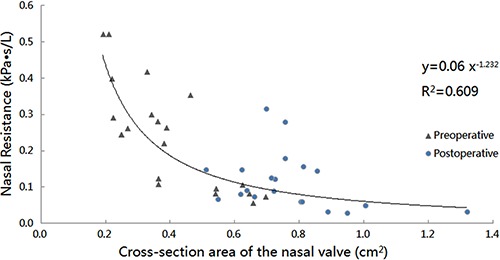
Pre- and postoperative unilateral nasal resistance *vs.* cross-sectional area at the nasal valve on the narrow side of nasal cavity.

The cross-sectional areas of the nasal valve increased at varying degrees among these patients. A significant postoperative improvement of nasal ventilation was observed in all patients. Notably, surgery corrected the nasal valve stenosis and achieved balanced ventilation in the bilateral nasal cavities. All factors contributing to nasal asymmetry can affect pressure, velocity, and airflow distributions within the nasal cavity (8). Our results indicated that the mean resistance in the narrow side of the nasal cavity was 0.241±0.14 Pa^.^s/mL preoperatively, which was significantly higher than that of postoperatively (0.111±0.07 Pa^.^s/mL; P<0.01; Table 5). Data confirmed that surgery reduced nasal resistance and thus improved nasal breathing. Recent research (20) showed that CFD could evaluate airflow features after nasal cavity ventilation expansion techniques. In that study, for preoperative obstructive sleep apnea hypopnea syndrome patients whose narrowest site was located in the nasal cavity, the nasal cavity expansion technique reduced the total nasal resistance. Although only three cases were reported in the study, their results were consistent with our current findings. As shown in Figure 7, we found that unilateral nasal resistance was correlated with the cross-sectional area at the nasal valve (correlation coefficient R^2^=0.609). Clearly, any increase in the cross sectional area would reduce nasal resistance.

**Figure 8:**
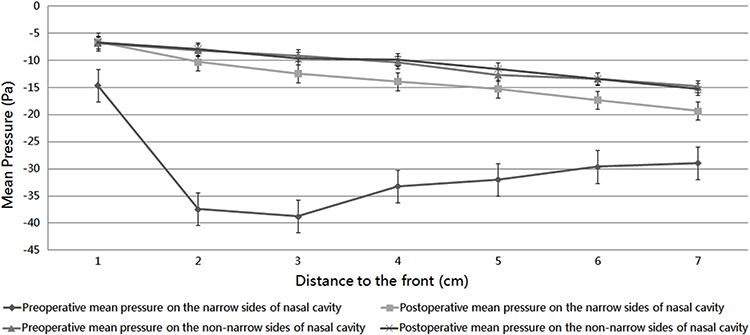
Pre- and postoperative mean pressure on representative cross-sections of the narrow and non-narrow sides of the nasal cavity.

Postoperatively, the pressure in the narrow side of the nasal cavity decreased and was comparable to the non-narrow side (Figure 8). This result shows that surgery can not only improve local nasal structure and resistance, but also provide bilateral airflow pressure uniformity.

Our study has confirmed that numerical simulation can provide a direct and objective basis for the assessment of pre- and postoperative anterior nasal cavity stenosis airflow, and also allows for a detailed description of the biophysics of nasal airflow. The anterior portion of the inferior turbinate, particularly the nasal valve area, has the most significant impact on the airflow gradient distribution. For anterior nasal cavity stenosis, CFD can be used to analyze postoperative nasal airflow features. Clearly, removal of the stenosis is essential for restoring nasal ventilation and obtain balanced nasal functions. The number of patients involved in the present study was quite small and the data might not be representative of larger populations. Future studies with larger samples are needed to prospectively validate our results.
